# Feasibility and Safety of Left Bundle Branch Pacing for Advance Aged Patients: A Multicenter Comparative Study

**DOI:** 10.3389/fcvm.2021.661885

**Published:** 2021-07-27

**Authors:** Zhongyuan Ren, Binni Cai, Songyun Wang, Peng Jia, Yang Chen, Rong Guo, Hailing Li, Jun Zhang, Jing Xiong, Jingying Zhang, Haotian Yang, Xiang Li, Yawei Xu, Xueying Chen, Dongdong Zhao

**Affiliations:** ^1^Department of Cardiology, Shanghai Tenth People's Hospital, Tongji University School of Medicine, Shanghai, China; ^2^Department of Cardiology, Xiamen Cardiovascular Hospital, Xiamen University, Xiamen, China; ^3^Department of Cardiology, Renmin Hospital of Wuhan University, Wuhan University School of Medicine, Wuhan, China; ^4^Department of Cardiology, National Clinical Research Center for Interventional Medicine, Shanghai Institute of Cardiovascular Diseases, Zhongshan Hospital of Fudan University, Shanghai, China

**Keywords:** physiological pacing, left bundle branch pacing, elderly, symptomatic bradycardia, safety

## Abstract

**Background:** Left bundle branch pacing (LBBP) has been shown to be a safe and effective means to achieve physiological pacing. However, elderly patients have increased risks from invasive procedures and the risk of LBBP in elderly patients is not known. We aimed to investigate the safety and efficacy of LBBP in elderly patients >80 years of age.

**Methods:** From December 2017 to June 2019, 346 consecutive patients with symptomatic bradycardia, 184 patients under 80 years of age and 162 over 80 years, were included and underwent LBBP. The safety and prognosis of LBBP were comparatively evaluated by measured pacing parameters, periprocedural complications, and follow-up clinical events.

**Results:** Compared with the younger, the elderly group had worse baseline cardiac and renal function. LBBP was achieved successfully in both groups with comparable fluoroscopic time and paced QRS duration (110.0 [102.0, 118.0] ms for the young vs. 110.0 [100.0, 120.0] ms for the elderly, *P* = 0.874). Through a follow-up of 20.0 ± 6.1 months, pacing parameters were stable while higher threshold and impedance were observed in the elderly group. In the evaluation of safety, overall procedure-related complication rates were comparable (4.4 vs. 3.8%, young vs. elderly). For prognosis, similar rates of major adverse cardiocerebrovascular events (7.1 vs. 11.9%, young vs. elderly) were observed.

**Conclusions:** Compared to younger patients, LBBP could achieve physiological pacing in patients over 80 with comparable midterm safety and prognosis. Long-term safety and benefits of LBBP, however, necessitate further evaluation.

## Introduction

Physiological pacing—imitating the normal cardiac conduction pathway—has long been put forward as a means of restoring atrioventricular synchrony. This concept has been historically redefined since the first His-bundle pacing attempt to achieve ventricular synchrony in 2000 (1). Thereafter, a growing body of evidence shows the efficacy of His-bundle pacing (2, 3). However, most studies utilize advanced pacemakers for a limited population (4–6), which cannot be generalized to patients requiring a more cost-effective therapy. In addition, early battery depletion often occurred as a result of the elevated pacing threshold, impeding the application of His-bundle pacing (7). Su et al. optimized the technique by pacing at the distal His-bundle or even closer to the left bundle branch (LBB), presenting a narrow QRS with steady pacing parameters (8). Furthermore, in 2017 they reported the first case of LBB pacing (LBBP) that safely corrected the LBB block in a heart failure patient and showed steady pacing parameters during follow-up (9). Based on current evidence, LBBP seems to be a safe and effective alternative to conventional pacing (10–12).

As the conductive pathway degenerates, aged patients had a higher incidence of symptomatic bradycardia, which can only be corrected by implantation of a pacemaker. Nevertheless, elderly patients have distinctive features compared with the general population: more tortuous veins, lower BMI, and lower cardiac mass (13). These differences increase the potential risks of the implantation procedure. Additionally, comorbidities like hypertension, ischemic heart disease, and chronic renal disease (14) are pervasive in the elderly population, which could further worsen the prognosis for pacemaker implantation. Although, LBBP is a promising approach, inevitable transseptal lead fixation and mapping of His and LBB potential would presumably pose a higher risk for complications. To our knowledge, no current study has investigated the feasibility and safety of LBBP specifically in an advanced age population.

Therefore, our multicenter comparative study was designed to observe the feasibility and safety of LBBP in patients over age 80 compared to younger patients.

## Materials and Methods

### Study Sample

From October 2018 to June 2019, 346 consecutive patients from Shanghai Tenth people's Hospital, Zhongshan Hospital of Fudan University, and Xiamen Cardiovascular Hospital admitted with symptomatic bradycardia were included. Symptomatic bradycardia was defined as ECG recorded sick sinus syndrome, atrial fibrillation with long R-R interval, high grade, 2nd and 3rd degree atrial ventricular (AV) block, which were in accordance with the 2013 ESC guidelines (15). Patients were excluded if they indicated and received cardiac resynchronization therapy (CRT) or implantation of implantable cardioverter-defibrillator (ICD). Written forms of consent were acquired from every patient before the procedure. Our study complied with the Declaration of Helsinki and was approved by the local ethical committee of Shanghai Tenth People's Hospital.

### LBBP Procedure

#### Location and Fixation

**Figure 1:**
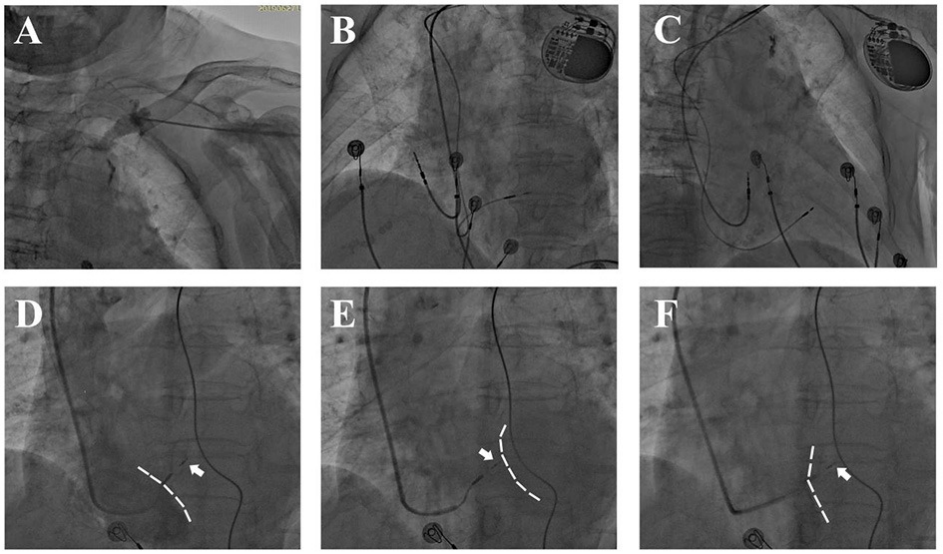
Fluoroscopy during LBBP. **(A)** Venous angiography after puncture showed a tortuous left subclavian vein. **(B,C)** Postprocedural fluoroscopy showed a fixed 3,830 lead in the IVS and atrial lead in right atrial appendage. **(D–F)** Lead depth measured by the relative position of 3,830 electrode (*white arrow*) and IVS during screwing. **(D,E)** Angiography *via* 8.5 F puncture sheath delineated RV silhouette [**(D)**, *dashed white line*] while delayed contrast delineated LV silhouette [**(E)***, dashed white line*]. **(F)** Angiography *via* C315 sheath showed RV side of IVS (*dashed white line*).

Details of His-bundle pacing procedure was reported in a previous study (16). Through, the left subclavian vein or axillary vein (Figure 1A), an 8.5 F sheath was placed after a fixed curved sheath (C315 His, Medtronic) distally advanced beyond the tricuspid annulus (Figures 1B,C). A Select Secure™ lead (model 3830, 69 CM, Medtronic, Minneapolis, MN, USA) was then cannulated to locate the His-bundle by capturing the His-bundle potential displayed on electrocardiogram (ECG, Bard recorder, Bard Electrophysiology Laboratory System, MA). Afterwards, under a right anterior oblique (RAO) 30° view, activation mapping was conducted 1 cm anterior to His-bundle to locate the eligible site—the proximal LBB, where left and right activations fuse incompletely and show a negative “W” waveform on lead V1. Then, the electrode was manipulated perpendicularly to the interventricular septum (IVS) and screwed clockwise until it reached the left ventricular (LV) subendomyocardium. During the procedure, the duration from the pacing signal to the peak of R wave (on V4-V6 lead) is measured as pacing to left ventricular activation time (p-LVAT), It reflects the activation time of the lateral wall of the left ventricle. An eligible site of left bundle capture was confirmed if selective LBBP was demonstrated by ECG, if p-LVAT shortened abruptly >10 ms through increasing pacing output, or if p-LVAT stayed shortest and stable at the site (17, 18).

#### Procedural Safety

**Figure 2:**
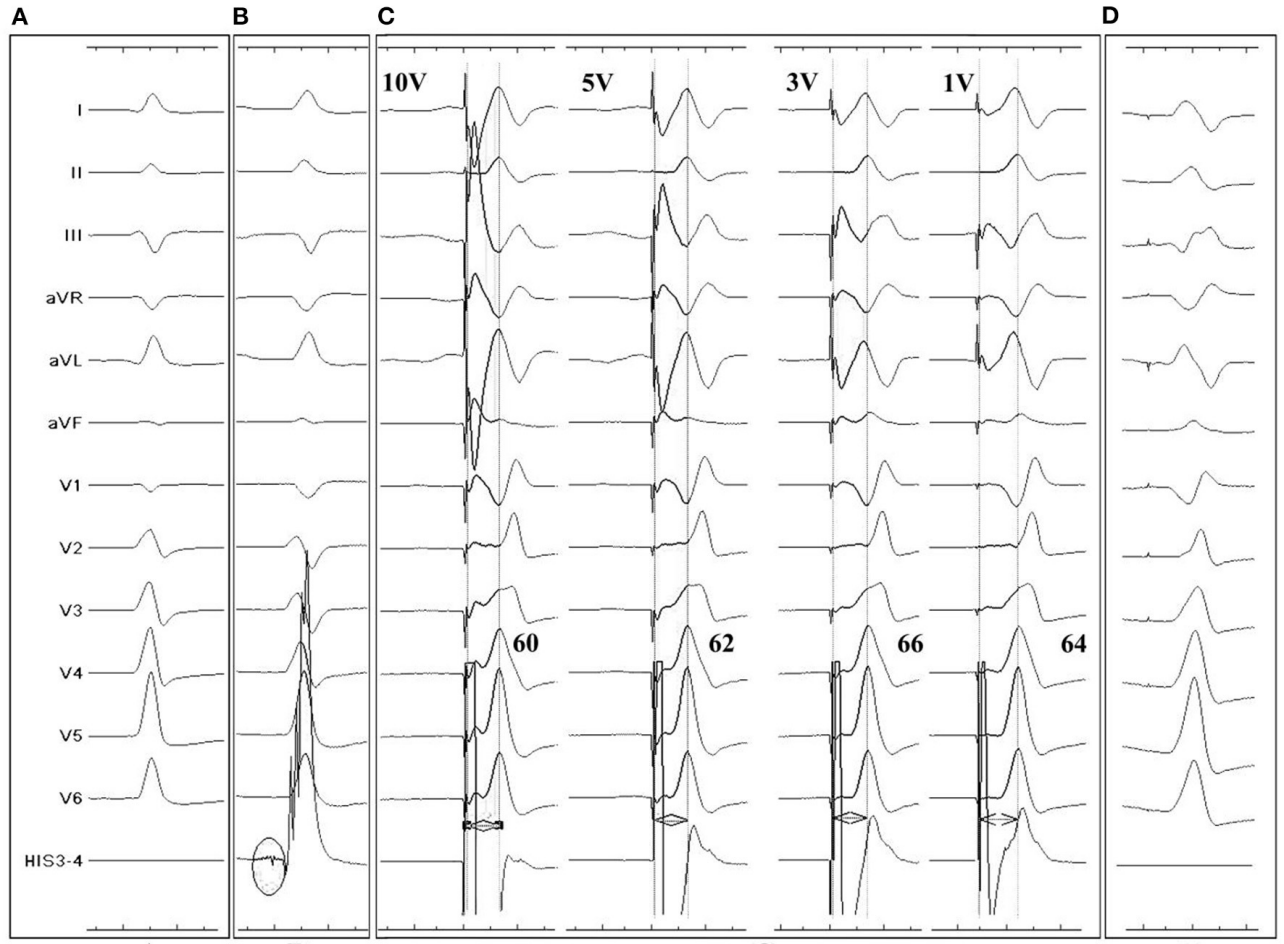
Twelve-lead and intracardiac electrocardiograms (ECG) during LBBP for a 96-year-old III° AVB patient. **(A):** Intrinsic rhythm showed a QS morphology in V1 lead. **(B)** LBB potential recorded by pacing tip (*black circle*) when the lead reached LV endocardium. **(C)** p-LVAT measured by unipolar pacing after fixation. Under different pacing output, QRS showed similar RBB block pattern and p-LVAT stayed short, indicating capture of LBB. **(D)** Postprocedural ECG recording. A QR pattern was shown in V1 lead. Paced QRS duration was similar to intrinsic.

To ensure safe and stable pacing, pacing thresholds, sensing, and impedance were measured. The intrinsic and paced QRS duration and p-LVAT were measured and optimized to mimic physiological conduction (Figure 2).

In order to prevent perforation and optimize fixation, the lead depth in the IVS was approximated under digital subtraction angiography (DSA) by injecting contrast media *via* the puncture sheath and C315 sheath (Figures 1D–F). Firstly, the angiography would delineate the silhouette of the right ventricle (RV) while delayed contrast delineated the LV silhouette. The contrast *via* C315 sheath would retain at the RV side of IVS, and the distance between the retention of contrast and the tip of pacing lead was measured as the lead depth in the IVS. For patients with AV block or complete LBB block, a temporary pacemaker was placed prior to LBBP in case of complete AV block resulting from injury of the His-bundle or proximal LBB.

#### Pacing Parameters and Device Programming

Paced QRS duration was routinely measured from the end of pacing signal to the end of QRS complex under bipolar pacing at acceptable pacing output, with a pulse width of 0.42 ms during the procedure. Pacing output and AV delay were adjusted individually to achieve optimal QRS morphology before discharge. During follow-up, pacing threshold, sense, impedance, and AV delay were routinely measured, with a pulse width of 0.40 ms.

### Safety and Prognosis Evaluation

Safety was evaluated by periprocedural and follow-up safety events, including lead-related complications such as lead failure, fracture, and dislodgement, pocket-related complications such as pocket hematoma and infection, and procedure-related complications such as pneumothorax, pericardial effusion, and cardiac tamponade.

Prognosis was evaluated by all-cause mortality, rehospitalization due to cardiovascular disease (CVD), and major adverse cardiocerebrovascular events (MACE) during follow-up. MACE was defined as the onset of severe cardiocerebrovascular events including acute myocardial infarction, acute decompensated heart failure, cardiac tamponade, malignant arrhythmia, stroke (infarction and hemorrhage), pacemaker reimplantation, and death due to CVD.

In the 1st, 3rd, and 12th month following LBBP procedure, patients were required to have outpatient or inpatient follow-up (if they were immobilized). Comprehensive medical histories were taken and physical examinations were conducted by experienced cardiologists. Device programming was required at every follow-up, and 24-h Holter and transthoracic echocardiography (TTE) were performed when physicians considered them necessary.

### Statistical Analysis

Continuous parameters were described as a mean ± standard deviation (SD) if they conformed to normal distribution, while those without a normal distribution were presented as the median and interquartile ranges (IQR). The *p*-value was generated from two sample *t*-tests or a Mann-Whitney test according to the equality of variance, or singed-rank test if a normal distribution was not presented. Repeated measures analysis of variance was applied to analyze the repeated measurements of pacemaker, electrocardiographic, and echocardiographic parameters. Categorical variables were described as percentages (%) and *p*-values were analyzed with χ^2^ tests or Fisher exact tests (when theoretical frequency was lower than 5). The incidence of procedure related complications, MACE, and CVD hospitalization were analyzed using Kaplan-Meier estimate, with *P*-value generated from Log-Rank test. A two-sided *P*-value of <0.05 was considered statistically significant. SAS 9.4 software (SAS Institute Inc., Cary, NC, USA) was used to conduct the analysis.

## Results

### Sample Characteristics

The median age of the younger and the elderly groups were 73.0 [65.0, 77.0] years and 84.0 [82.0, 87.0] years, respectively. The proportion of male and female patients were similar. The indications were similar between groups. Compared with the younger group, the elderly had significantly deteriorated renal function (estimated glomerular filtration fraction (eGFR) 65.2 ± 26.6 vs. 89.1 ± 30.8 ml/min/1.73m^2^, *P* < 0.001). Although, the cardiac function evaluated by left ventricular ejection fraction (LVEF) (*P* = 0.275) was similar, the level of N-terminal pro-brain natriuretic peptide (NT-proBNP) was higher (the elderly group vs. the younger group, 1076.0 [324.0, 2513.0] vs. 273.7 [100.6, 752.7] pg/ml, *P* < 0.001) and cardiac function evaluated by New York Heart Association (NYHA) grading was worse in the elderly group (*P* < 0.001). In addition, the prevalence of heart failure was higher in the elderly group (25.2 vs. 11.5%, *P* = 0.001). Of note, although, there was an unequal distribution of IVS thickness between the younger and the elderly group measured by TTE (*P* = 0.010 generated by Wilcoxon sign rank test), such a difference was too small in value to be clinically significant. Other comorbidities and medications were similar. Detailed information is listed in Table 1.

**Table 1:** Baseline characteristics of both young and elderly patients.

**Variables**	**Overall *N* = 341**	**Young (<80) *N* = 182**	**Elderly (≥80) *N* = 159**	***P*-value**
Age, yrs	80.0 [71.0, 84.0]	72.0 [65.0, 77.0]	84.0 [82.0, 87.0]	**<0.001**
Gender (male), *n* (%)	173 (50.7)	94 (51.7)	79 (49.7)	0.717
IVS thickness, mm	10.0 [10.0, 11.0]	10.0 [10.0, 11.0]	10.0 [10.0, 11.0]	**0.010**
LVEF, %	60.0 [55.0, 62.0]	60.0 [57.0, 62.0]	60.0 [55.0, 62.0]	0.275
NT-proBNP, pg/ml	539.4 [181.3, 1576.0]	273.7 [100.6, 752.7]	1076.0 [324.0, 2513.0]	**<0.001**
eGFR, ml/min/1.73m^2^^*^	79.2 ± 31.4	89.1 ± 30.8	65.2 ± 26.6	**<0.001**
NYHA, *n* (%)				**0.003**
IV	23 (6.7)	9 (5.0)	14 (8.8)	
III	49 (14.4)	20 (11.0)	29 (18.2)	
II	95 (27.7)	42 (23.1)	53 (33.3)	
I	174 (50.7)	111 (61.0)	63 (39.6)	
Indications, *n* (%)				0.887
SSS	127 (37.2)	72 (39.6)	55 (34.6)	
AF with long R-R interval	53 (15.5)	27 (14.8)	26 (16.4)	
AVB^+^	147 (43.1)	75 (41.2)	72 (45.3)	
Lead revision	2 (0.6)	1 (0.6)	1 (0.6)	
Battery depletion	12 (3.5)	7 (3.9)	5 (3.1)	
**Medical history**, ***n*****(%)**				
Heart failure	61 (17.9)	21 (11.5)	40 (25.2)	**0.001**
AF/AFL	101 (29.1)	51 (28.0)	49 (31.0)	0.788
DCM	5 (1.5)	3 (1.7)	2 (1.3)	1.000
HCM	12 (3.5)	5 (2.8)	7 (4.4)	0.408
Coronary artery disease	93 (27.3)	44 (24.2)	49 (30.8)	0.170
Hypertension	253 (74.2)	130 (71.4)	123 (77.4)	0.212
Diabetes mellitus	85 (24.9)	47 (25.8)	38 (23.9)	0.682
**Medications**, ***n*****(%)**				
Antiplatelet agents	87 (25.5)	41 (22.5)	46 (29.0)	0.389
Oral anticoagulants	31 (9.1)	16 (8.8)	15 (9.4)	0.837

*Continuous variables are described as mean ± SD or median with IQR, while categorical variables are presented as percentages (%). AF, denotes atrial fibrillation; AFL, atrial flutter; AVB, atrial ventricular block; BMI, body mass index; DCM, dilated cardiomyopathy; eGFR, estimated glomerular filtration fraction; HCM, hypertrophic cardiomyopathy; IVS, interventricular septum; LVEF, left ventricular ejection fraction; NT-proBNP, N-terminal pro brain natriuretic peptide; NYHA, New York Heart Association grading of cardiac function; SSS, sick sinus syndrome*.

* *eGFR was calculated by MDRD formula*.

+ *AVB includes high grade AVB, II° AVB Mobitz type 2 and III° AVB. The bold value indicates significant P-value (P < 0.05)*.

### Periprocedural Measurements

**Figure 3:**
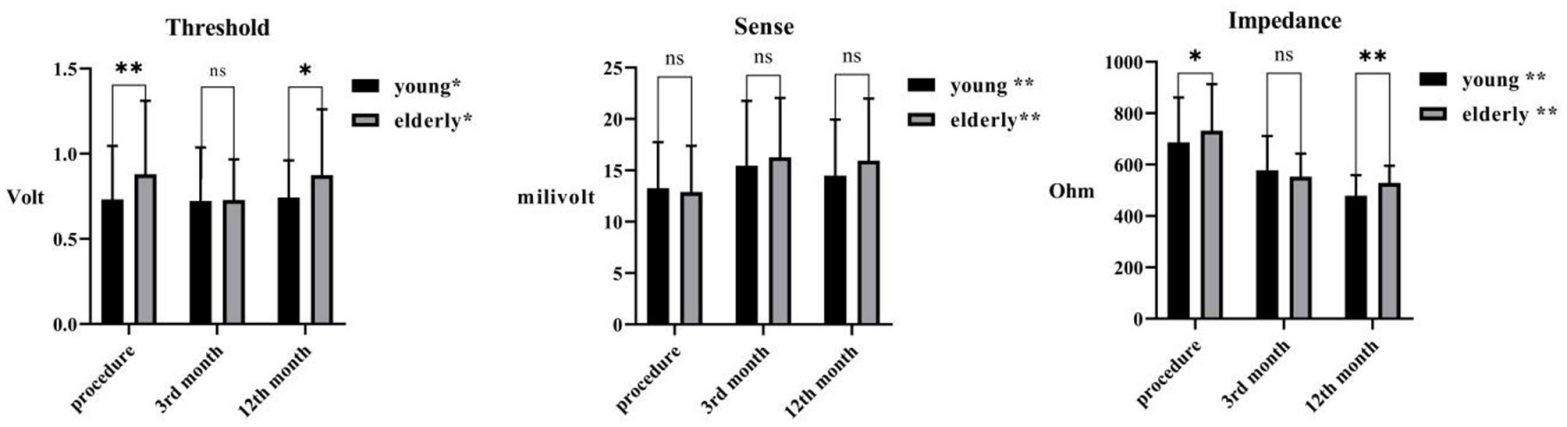
Pacing parameters during LBBP in the 3rd and 12th month after implantation between young and elderly groups. * and ** indicates statistical significance, *p* < 0.05 and 0.01, respectively, and ns denotes non-significance.

LBBP was achieved in all 346 patients. The fluoroscopic time and dosage were similar between groups. Paced QRS duration (110.0 [102.0, 118.0] vs. 110.0 [100.0, 120.0] ms, *P* = 0.874) were shortened and comparable between groups. After lead fixation, lead sense was similar (13.3 ± 4.5 vs. 12.9 ± 4.5 mV), while higher pacing threshold (0.73 ± 0.31 vs. 0.87 ± 0.43 V, *P* < 0.001) and impedance (686.3 ± 175.0 vs. 732.1 ± 180.5 ohms, *P* < 0.01) were observed in the elderly group (Figure 3). Of note, a higher proportion of temporary pacemaker implantation prior to LBBP was observed in the elderly group (18.2 vs. 8.8%). Details are presented in Table 2.

**Table 2:** Procedural details of LBBP.

**Parameters**	**Overall *N* = 341**	**Young (<80) *N* = 182**	**Elderly (≥80) *N* = 159**	***P*-value**
Fluoroscopic time, min	10.3 [7.4, 16.2]	11.2 [7.7, 16.6]	9.65 [7.4, 15.3]	0.311
Fluoroscopic dosage, mGy	135.9 [85.9, 246.8]	138.5 [96.2, 255.8]	130.0 [76.5, 226.0]	0.227
**Preprocedural measurements**				
QRS duration, ms	104.0 [94.0, 136.5]	102.0 [93.0, 137.0]	106.0 [94.0, 136.0]	0.600
LBB block, *n* (%)	25 (7.4)	11 (6.1)	14 (8.8)	0.329
RBB block, *n* (%)	38 (11.2)	21 (11.6)	17 (10.7)	0.804
Temporary pacemaker, *n* (%)	45 (13.2)	16 (8.8)	29 (18.2)	**0.010**
**Intraprocedural measurements**				
Paced QRS duration, ms	110.0 [102.0, 118.0]	110.0 [102.0, 118.0]	110.0 [100.0, 120.0]	0.874
LBB potential recorded, *n* (%)	164 (54.3)	86 (58.9)	78 (50.0)	0.121
p-LVAT, ms	72.0 [66.0, 80.0]	72.0 [66.0, 80.0]	70.0 [64.0, 78.0]	0.299

*LBB, denotes left bundle branch; p-LVAT, pacing to left ventricle activation time; RBB, right bundle branch. The bold value indicates significant P-value (P < 0.05)*.

### Evaluation of Safety and Prognosis

Over a 20.0 ± 6.1 month period, five (1.5%) patients were lost to follow-up. During follow-up, there was a rise of pacing threshold in the elderly group (*P* < 0.01 in both groups), which was higher than that of the younger group in the 12th month (young vs. elderly 0.74 ± 0.22 vs. 0.87 vs. 0.39 V, *P* < 0.01). The sensing was risen in both groups, while the it was comparable between groups. And the impedance was decreased in both groups (*P* < 0.01 in both groups), although, it was higher in the elderly group (young vs. elderly, 479.2 ± 80.0 vs. 528.3 ± 66.7 ohms, *P* < 0.001). Such minor changes of pacing parameters indicates that the lead has a stable performance through a mid-term follow-up.

**Figure 4:**
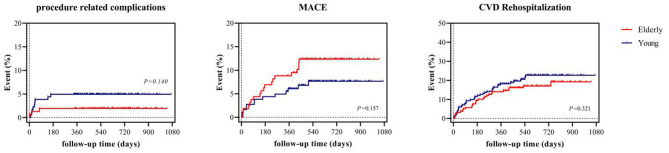
Survival analysis of procedure related complications, MACE, and rehospitalization due to CVD. MACE denotes major adverse cardiovascular event, CVD cardiovascular disease.

In terms of safety, the incidence of procedure-related complications was similar in both the young (4.4%) and elderly group (3.8%). The overall MACE incidence was comparable in the elderly group (young 7.1 vs. elderly 11.9%, *P* = 0.157). Notably, the incidence of cerebral infarction (0 vs. 3.1%, *P* = 0.050) and myocardial infarction (2.5 vs. 0%, *P* = 0.099) were non-significantly higher in the elderly group. In addition, a similar proportion of patients underwent rehospitalization due to CVD during follow-up (young 12.1 vs. elderly 13.8%, *P* = 0.321). Follow-up details are listed in Table 3 and survival analysis of procedure related complications, MACE, and rehospitalization due to CVD are demonstrated in Figure 4.

**Table 3:** Safety and prognosis between younger and elderly patients.

**Events**	**Overall *N* = 341**	**Young (<80) *N* = 182**	**Elderly (≥80) *N* = 159**	***P*-value**
**Procedure related complications**	14 (4.1)	8 (4.4)	6 (3.8)	0.140
Lead fracture, *n* (%)	0	0	0	1.000
Lead dislodgement, *n* (%)	1 (0.3)	0	1 (0.6)	0.946
Atrial perforation, *n* (%)	1 (0.3)	0	1 (0.6)	0.946
Ventricular perforation, *n* (%)	0	0	0	1.000
Pocket hematoma, *n* (%)	3 (0.9)	2 (1.1)	1 (0.6)	0.565
Pocket infection, *n* (%)	4 (1.2)	4 (2.2)	0	0.169
Incision algesia, *n* (%)	1 (0.3)	1 (0.5)	0	0.946
Pericardial effusion, *n* (%)	5 (1.5)	1 (0.5)	4 (2.5)	0.291
**MACE**	32 (9.4)	13 (7.1)	19 (11.9)	0.157
Acute myocardial infarction, *n* (%)	4 (1.2)	0	4 (2.5)	0.099
Acute heart failure, *n* (%)	15 (4.4)	9 (4.9)	6 (3.8)	0.794
Ventricular fibrillation, *n* (%)	1 (0.3)	1 (0.5)	0	0.946
Cerebral infarction, *n* (%)	5 (1.5)	0	5 (3.1)	0.050
Subdural hemorrhage, *n* (%)	1 (0.3)	1 (0.5)	0	0.946
Pacemaker reimplantation, *n* (%)	1 (0.3)	1 (0.5)	0	0.946
Cardiac tamponade, *n* (%)	1 (0.3)	0	1 (0.6)	0.946
Death due to CVD, *n* (%)	4 (1.2)	1 (0.5)	3 (1.9)	0.522
**Rehospitalization due to CVD**, ***n*****(%)**	44 (12.9)	22 (12.1)	22 (13.8)	0.321
**All-cause mortality**, ***n*****(%)**	9 (2.6)	3 (1.6)	6 (3.8)	0.377

*CVD, denotes cardiovascular disease; MACE, major adverse cardiocerebrovascular events*.

**Figure 5:**
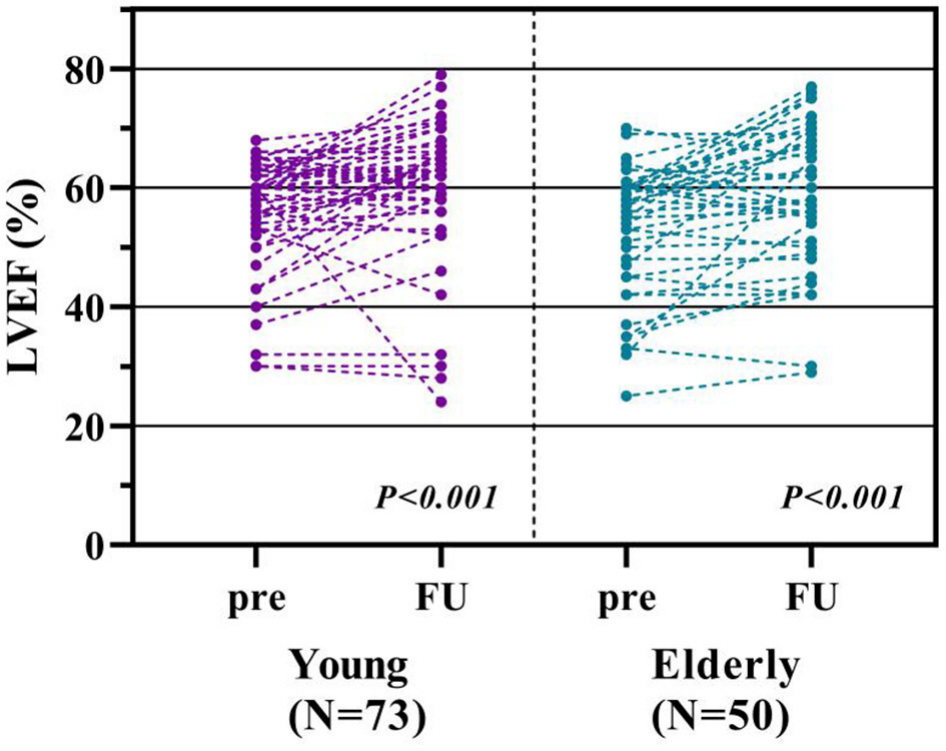
Comparison of echocardiographic measurements. Figure from the left to the right showed the comparison of preprocedural and the 12th month measurements of left ventricular ejection fraction (LVEF) between young and elderly groups.

Cardiac function measured by TTE were collected and compared in 73 younger and 50 elderly patients. Statistic significant improvement of LVEF was observed in both the young (*P* < 0.001) and elderly groups (*P* < 0.001). Only one younger patient had worsening cardiac function (LVEF dropped from 60 to 24%) resulting from pneumonia-induced acute heart failure (Figure 5).

## Discussion

Our multicenter comparative study compared the profiles of 159 elderly patients aged over 80 with 182 younger patients with symptomatic bradycardia who underwent LBBP. Our findings suggest that physiological pacing *via* LBBP can be performed in elderly patients without increasing the risk of complications and that midterm prognosis of elderly patients undergoing LBBP was comparable with the younger patient group.

Population aging is a major issue, with one report estimating over 150 million Chinese citizens will be over 80 by 2050 (19). Elderly patients should be considered as a special community, as they have more co-morbidities and worse prognosis. Especially in the consideration of pacemaker implantation, elderly patients had more tortuous veins, lower BMI, and lower cardiac mass, which accounts for the higher risk of complications such as pneumothorax, lead dislodgement, perforation, and loss of capture (20). Therefore, investigating the safety and prognosis of pacemaker implantation in the advanced aged population is of great importance.

LBBP is a novel and feasible pacing maneuver to achieve physiological pacing. LBBP requires the capture of left bundle branch potential to mimic the normal electric conduction. In our multicenter study, LBB potential is recorded in 54.3% of the population, and 58.9% of the younger group and 50.0% of the elderly group, respectively. The capture of left conduction system could be hard, as most studies on LBBP reported that the ratio of LBB potential capture ranged between 50 and 80% (3, 12, 21, 22). An animal study has confirmed that positioning the lead deep enough to the left septal subendomyocardium could easily capture the left conduction system (23). Therefore, based on our findings, we believe that in most situations the capture of the left conduction system is mostly dependent on lead manipulation rather than age and condition of patients. Besides, several clinical evidences validated that LBBP could be achieved safely (3, 10, 12, 24). However, most studies have failed to evaluate the efficacy in the advanced elderly population. In our evaluation of safety, through a follow-up of 20.0 ± 6.1 months, the incidence of overall safety events was low in both groups and similar to that of previous studies (21). Among complications related to LBBP, lead-related complications rarely occur. Chen et al. reported two complications in 612 patients (24) and Su et al. reported two cases of lead dislodgement in 632 patients (10), which are similar to the incidence in our study. Since Huang et al. (16) published and standardized the LBBP maneuver and criteria (18), complications like lead dislodgement have been rarely reported in an experienced center.

Specifically, elderly patients undergoing LBBP were at a higher risk of perforation and should be independently considered. One previous study has shown that ventricular perforation is correlated with several factors during conventional pacing, including the use of temporary pacemakers, use of steroids, use of helical screw leads, BMI of <20, and old age (13). Most importantly, their study indicates that a thinning of the cardiac wall in the elderly population and excessive leads in the RV were the major risk factors contributing to perforation. In the consideration of LBBP, multiple leads were routinely used including one or two active fixation 3,830 leads and sometimes temporary pacing lead, which could presumably pose a higher risk of complication, especially when LBBP was performed in the elderly population. In the present study, four out of five cases of periprocedural pericardial effusion occurred in the elderly group and one case of cardiac tamponade occurred requiring pericardiocentesis, which failed to reach a statistical significance. Compared with previous studies on LBB and His-bundle pacing, the incidence of perforation ranged from 0 to 3%, (10, 12, 24) which was relatively low and comparable with ours.

Collectively, we believe the overall safety of LBBP in the elderly is acceptable in an experienced center. However, we acknowledge that the incidence of complications was still too low to detect the significance; larger scaled studies are warranted to provide stronger evidence on safety in the elderly population. Based on our experience, LBBP should be performed with extra caution in patients with advanced age, while assessment of lead depth by angiography could help prevent perforation. We recommend assessment of lead depth with the following criteria: (1) Unipolar pacing impedance at the distal tip should be > 500 ohms (sharp decrement indicates perforation into LV); (2) Once LBB potential has been recorded and pacing parameters are acceptable, screwing should be immediately stopped; and (3) Under DSA, we judged lead depth by continuously injecting contrast (Figures 1D–F). In addition, when retracting the delivery sheath, a rebound of the distal portion of the lead should be monitored to confirm stable fixation (16).

Last but not least, the benefits of LBBP in patients over the age of 80 was also comparable with the younger population. LBBP could achieve physiological conduction, mechanical synchrony, and correct LBB block (3, 6, 25, 26), and presumably could improve the outcomes of patients with bradycardia. Our multicenter study showed that LBBP in elderly patients could indeed achieve comparable shortening of QRS duration and improvement of cardiac function with the younger group, and such results were in accord with the previous published studies (3, 12, 26). However, although, not statistically significant, there was a tendency of higher incidence of MACE in the elderly group including acute myocardial infarction and cerebral infarction. We believe such a tendency resulted from the high rate of comorbidities in the elderly population. Therefore, we believe that LBBP could correct bradycardia with better electrical and mechanical synchrony in elderly patients, but the benefits should not be overestimated.

## Limitation

Our results should be interpreted with caution. First, our results cannot be extrapolated to patients who undergo pacemaker implant for reasons other than symptomatic bradycardia. Second, we aimed to compare the performance of LBBP between two age groups, while a comparison of LBBP with conventional RV pacing in the elderly population could better validate the benefits and risks of LBBP. Well-designed, large-scaled, comparative studies are required to further illustrate the safety and efficacy of LBBP in the elderly population. In addition, although, the sample size was considerable and the follow-up period was over 1 year, it was still too short to detect a late difference between groups. Studies of a larger scale and with longer follow-up periods are necessary to validate the long-term safety and benefits of LBBP.

## Conclusions

Compared to the younger group, LBBP could be achieved in patients over 80 years old with symptomatic bradycardia, and comparable mid-term safety and prognosis can be observed. Long-term safety and benefits of LBBP still require further evaluation.

## Data Availability Statement

The raw data supporting the conclusions of this article will be made available by the authors, without undue reservation.

## Ethics Statement

The studies involving human participants were reviewed and approved by ethics committee of Shanghai Tenth People's Hospital. The patients/participants provided their written informed consent to participate in this study. Written informed consent was obtained from the individual(s) for the publication of any potentially identifiable images or data included in this article.

## Author Contributions

DZ, XC, and SW contributed to the interpretation of data for the work. ZR and BC contributed to drafting the work. PJ, YC, JuZ, JiZ, HY, and XL contributed to the acquisition of data. RG, HL, and JX contributed to the analysis and revision of the work. DZ, XC, and YX contributed to the conception of the work. All authors contributed to the article and approved the submitted version.

## Conflict of Interest

The authors declare that the research was conducted in the absence of any commercial or financial relationships that could be construed as a potential conflict of interest.

## Publisher's Note

All claims expressed in this article are solely those of the authors and do not necessarily represent those of their affiliated organizations, or those of the publisher, the editors and the reviewers. Any product that may be evaluated in this article, or claim that may be made by its manufacturer, is not guaranteed or endorsed by the publisher.

## References

[1] Deshmukh P Casavant DA Romanyshyn M Anderson K. Permanent, direct His-bundle pacing: a novel approach to cardiac pacing in patients with normal His-Purkinje activation. Circulation. (2000) 101:869–77. 10.1161/01.CIR.101.8.869 10694526

[2] Huang W Zhou X Ellenbogen KA. Pursue physiological pacing therapy: A better understanding of left bundle branch pacing and left ventricular septal myocardial pacing. Heart Rhythm. (2021). 10.1016/j.hrthm.2021.05.013. [Epub ahead of print]. 33992731

[3] Huang W Su L Wu S Xu L Xiao F Zhou X. Long-term outcomes of His bundle pacing in patients with heart failure with left bundle branch block. Heart. (2019) 105:137–43. 10.1136/heartjnl-2018-313415 30093543

[4] Huang W Su L Wu S Xu L Xiao F Zhou X. Benefits of permanent His bundle pacing combined with atrioventricular node ablation in atrial fibrillation patients with heart failure with both preserved and reduced left ventricular ejection fraction. J Am Heart Assoc. (2017) 6:e005309. 10.1161/JAHA.116.005309 28365568 PMC5533020

[5] Sharma PS Naperkowski A Bauch TD Chan JYS Arnold AD Whinnett ZI. Permanent His bundle pacing for cardiac resynchronization therapy in patients with heart failure and right bundle branch block. Circ Arrhythm Electrophysiol. (2018) 11:e006613. 10.1161/CIRCEP.118.006613 30354292

[6] Wu S Su L Vijayaraman P Zheng R Cai M Xu L. Left bundle branch pacing for cardiac resynchronization therapy: non-randomized on-treatment comparison with his bundle pacing and biventricular pacing. Can J Cardiol. (2021) 37:319–28. 10.1016/j.cjca.2020.04.037 32387225

[7] Sharma PS Dandamudi G Naperkowski A Oren JW Storm RH Ellenbogen KA. Permanent His-bundle pacing is feasible, safe, and superior to right ventricular pacing in routine clinical practice. Heart Rhythm. (2015) 12:305–12. 10.1016/j.hrthm.2014.10.021 25446158

[8] Su L Wu S Wang S Wang Z Xiao F Shan P. Pacing parameters and success rates of permanent His-bundle pacing in patients with narrow QRS: a single-centre experience. Europace. (2019) 21:763–70. 10.1093/europace/euy281 30561576

[9] Huang W Su L Wu S Xu L Xiao F Zhou X. A novel pacing strategy with low and stable output: pacing the left bundle branch immediately beyond the conduction block. Can J Cardiol. (2017) 33:1736.e1731–3. 10.1016/j.cjca.2017.09.013 29173611

[10] Su L Wang S Wu S Xu L Huang Z Chen X. Long-term safety and feasibility of left bundle branch pacing in a large single center study. Circ Arrhythm Electrophysiol. (2021) 14:e009261. 10.1161/CIRCEP.120.009261 33426907

[11] Huang W Wu S Vijayaraman P Su L Chen X Cai B. Cardiac resynchronization therapy in patients with non-ischemic cardiomyopathy using left bundle branch pacing. JACC Clin Electrophysiol. (2020) 6:849–58. 10.1016/j.jacep.2020.04.011 32703568

[12] Chen X Jin Q Bai J Wang W Qin S Wang J. The feasibility and safety of left bundle branch pacing vs. right ventricular pacing after mid-long-term follow-up: a single-centre experience. Europace. (2020) 22:ii36–44. 10.1093/europace/euaa294 33370799

[13] Mahapatra S Bybee KA Bunch TJ Espinosa RE Sinak LJ McGoon MD. Incidence and predictors of cardiac perforation after permanent pacemaker placement. Heart Rhythm. (2005) 2:907–11. 10.1016/j.hrthm.2005.06.011 16171740

[14] Antonelli D Freedberg NA Bushari LI Feldman A Turgeman Y. Permanent pacing in non-agenarians over 20-year period. Pacing Clin Electrophysiol. (2015) 38:48–53. 10.1111/pace.12499 25196677

[15] Brignole M Auricchio A Baron-Esquivias G Bordachar P Boriani G Breithardt OA. 2013 ESC Guidelines on cardiac pacing and cardiac resynchronization therapy: the Task Force on cardiac pacing and resynchronization therapy of the European Society of Cardiology (ESC). Developed in collaboration with the European Heart Rhythm Association (EHRA). Eur Heart J. (2013) 34:2281–329. 10.1093/eurheartj/eht150 23801822

[16] Huang W Chen X Su L Wu S Xia X Vijayaraman P. A beginner's guide to permanent left bundle branch pacing. Heart Rhythm. (2019) 16:1791–6. 10.1016/j.hrthm.2019.06.016 31233818

[17] Chen X Wu S Su L Su Y Huang W. The characteristics of the electrocardiogram and the intracardiac electrogram in left bundle branch pacing. J Cardiovasc Electrophysiol. (2019) 30:1096–101. 10.1111/jce.13956 31094058

[18] Wu S Chen X Wang S Xu L Xiao F Huang Z. Evaluation of the criteria to distinguish left bundle branch pacing from left ventricular septal pacing. JACC Clin Electrophysiol. (2021). 10.1016/j.jacep.2021.02.018. [Epub ahead of print]. 33933414

[19] Fang EF Scheibye-Knudsen M Jahn HJ Li J Ling L Guo H. A research agenda for aging in China in the 21st century. Ageing Res Rev. (2015) 24:197–205. 10.1016/j.arr.2015.08.003 26304837 PMC5179143

[20] Armaganijan LV Toff WD Nielsen JC Andersen HR Connolly SJ Ellenbogen KA. Are elderly patients at increased risk of complications following pacemaker implantation? A meta-analysis of randomized trials. Pacing Clin Electrophysiol. (2012) 35:131–4. 10.1111/j.1540-8159.2011.03240.x 22040168

[21] Li Y Chen K Dai Y Li C Sun Q Chen R. Left bundle branch pacing for symptomatic bradycardia: implant success rate, safety, and pacing characteristics. Heart Rhythm. (2019) 16:1758–65. 10.1016/j.hrthm.2019.05.014 31125667

[22] Sharma PS Dandamudi G Herweg B Wilson D Singh R Naperkowski A. Permanent His-bundle pacing as an alternative to biventricular pacing for cardiac resynchronization therapy: a multicenter experience. Heart Rhythm. (2018) 15:413–20. 10.1016/j.hrthm.2017.10.014 29031929

[23] Chen X Jin Q Li B Jia J Sharma PS Huang W. Electrophysiological parameters and anatomical evaluation of left bundle branch pacing in an in vivo canine model. J Cardiovasc Electrophysiol. (2020) 31:214–9. 10.1111/jce.14300 31778271

[24] Chen X Wei L Bai J Wang W Qin S Wang J. Procedure-related complications of left bundle branch pacing: a single-center experience. Front Cardiovasc Med. (2021) 8:645947. 10.3389/fcvm.2021.645947 33869306 PMC8044788

[25] Ye Y Wu S Su L Sheng X Zhang J Wang B. Feasibility and Outcomes of Upgrading to Left Bundle Branch Pacing in Patients With Pacing-Induced Cardiomyopathy and Infranodal Atrioventricular Block. Front Cardiovasc Med. (2021) 8:674452. 10.3389/fcvm.2021.674452 34195236 PMC8236829

[26] Cai B Huang X Li L Guo J Chen S Meng F. Evaluation of cardiac synchrony in left bundle branch pacing: insights from echocardiographic research. J Cardiovasc Electrophysiol. (2020) 31:560–9. 10.1111/jce.14342 31919928 PMC7027438

